# Population Density and Spatial–Temporal Niche Use of Ship Rats and Italian Wall Lizards on a Small Adriatic Island

**DOI:** 10.1002/ece3.74003

**Published:** 2026-08-03

**Authors:** Maja Perinović, Tomislav Gojak, Marko Glogoški, Luce Pavin, Hrvoje Čižmek, Duje Lisičić

**Affiliations:** ^1^ Division of Biology, Department of Animal Physiology, Faculty of Science University of Zagreb Zagreb Croatia; ^2^ Association Hyla Zagreb Croatia; ^3^ Marine Explorers Society 20 000 Leagues Zadar Croatia

**Keywords:** camera trapping, coexistence mechanisms, distance sampling, invasive mammals, island ecology, SECR

## Abstract

Introduced rats are major drivers of biodiversity loss on islands, yet density‐based assessments of their interactions with native reptiles remain scarce. On Mediterranean islands, long‐term rat‐lizard coexistence is facilitated by behavioral and morphological compensation mechanisms in lizards, but there are no studies on integrative assessments of population density and spatial and temporal niche partitioning. In this study, we quantified population density, spatial distribution, and diel activity patterns of ship rats ( *Rattus rattus* ) and Italian wall lizards (*Podarcis siculus*) on a small uninhabited Adriatic islet. Rat density was estimated using spatially explicit capture–recapture (SECR), while lizard density was assessed using line‐transect distance sampling. Camera trapping was used to examine the spatial and temporal niche use of all recorded terrestrial vertebrates. Rat density reached 32.1 individuals ha^−1^, and lizard density was estimated at 2345 individuals ha^−1^, both falling within the range reported for insular populations. Only three terrestrial vertebrates were detected on the islet: *R. rattus*, *P. siculus* , and European rabbits ( *Oryctolagus cuniculus* ), highlighting the structural simplicity of this island system. Rats and lizards showed broad spatial overlap across all habitat types, but pronounced temporal segregation, with rats being predominantly nocturnal and lizards strictly diurnal. Despite the presence of invasive rats, lizard density remained relatively high and within ranges reported for other insular systems, suggesting that temporal niche partitioning may facilitate coexistence under long‐term sympatry. However, studies across seasons and years are needed to determine whether this pattern is consistent. These results provide quantitative evidence of rat–lizard coexistence in a simplified Mediterranean island system and contribute to the global understanding of invasive species impacts under long‐term sympatry.

## Graphical abstract

We quantified population density and spatial–temporal niche use of ship rats ( *Rattus rattus* ) and Italian wall lizards (*Podarcis siculus*) on a small uninhabited Adriatic islet using spatially explicit capture–recapture, distance sampling, and camera trapping. Despite relatively high rat density, lizard density remained high, with near‐complete temporal segregation between nocturnal rats and diurnal lizards but broad spatial overlap, suggesting that temporal niche partitioning facilitates coexistence under long‐term sympatry in simplified Mediterranean island systems.

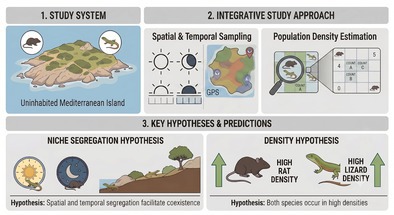

## 1 Introduction

Invasive species, usually introduced through human activities, can negatively impact native ecosystems (Jeschke et al. 2014). Indeed, invasive species are among the leading drivers of biodiversity loss worldwide, with particularly severe consequences in island ecosystems (Bellard et al. 2016). Owing to their isolation and high levels of endemism, island faunas are particularly vulnerable to invasive predators (Doherty et al. 2016). Among terrestrial vertebrates, mammals introduced through human activities have had particularly strong negative impacts on insular communities, often causing population declines, changes in habitat use, or local extinctions of native taxa (Courchamp et al. 2003).

Rats of the genus *Rattus* are among the most widespread and ecologically damaging invasive mammals on islands. Closely associated with humans, rats are now present on thousands of islands worldwide (Jones et al. 2008). Their invasion success is attributed to high reproductive output, dietary generalism, and behavioral flexibility, which enable them to exploit a wide range of habitats and resources. *Rattus* spp. are highly opportunistic omnivores that feed on fruits, seeds, vegetative plant material, and a variety of animal prey, primarily arthropods, whereas vertebrates such as birds and lizards generally constitute only a minor component of their diet (Shiels et al. 2013; Clapperton et al. 2019). Such ecological versatility increases the likelihood of spatial and temporal overlap with native fauna, thereby enhancing the potential for competitive or predatory interactions (Shiels et al. 2014; Harper and Bunbury 2015). As a result, introduced *Rattus* spp., together with invasive cats, have been identified as causal factors in 44% of modern bird, mammal, and reptile extinctions worldwide (Doherty et al. 2016). Several studies have documented negative effects of invasive rats on island lizards, including reduced abundance, altered population structure, shifts in habitat use, and behavioral or morphological changes following rat introduction or removal (Hoare et al. 2007; Towns et al. 2007; López‐Darias et al. 2024). Such effects are often attributed to predation on eggs, juveniles and adult lizards, competition for space and food, or indirect habitat‐mediated processes, including changes in vegetation structure and resource availability that may indirectly affect lizard populations (Towns et al. 2006; Shiels et al. 2014). Nocturnal or crepuscular lizards, such as geckos, are considered particularly vulnerable due to temporal overlap with rat activity, which may increase encounter rates and predation risk (Towns 1991; Hoare 2006).

Mediterranean islands are a global hotspot of biodiversity and endemism, and lizards represent a key component of their terrestrial ecosystems (Médail and Quézel 1999; Podnar et al. 2005). Lizards on Mediterranean islets often reach high population densities (Pérez‐Mellado et al. 2008) and exploit a range of microhabitats in response to different conditions (Bauwens et al. 1996). Insular populations of Mediterranean lizards may represent unique evolutionary units despite not being endemic species (Podnar Lešić 2005; Runemark et al. 2010). Although insular lizards populations may occur at high density, their total population sizes are often small due to the limited area available on islands. Consequently, they may be particularly sensitive to environmental changes, including the introduction of invasive mammals (Fernández‐Palacios et al. 2021).

It is estimated that the ship rat ( *Rattus rattus* ) was introduced to the Mediterranean between 4000 and 2000 years ago, subsequently colonizing numerous islands (McCormick 2003; Ruffino and Vidal 2010; Yu et al. 2022). Despite this long history of coexistence, relatively few studies have examined rat–lizard interactions in the region. A large‐scale study encompassing more than 159 islands in the western Mediterranean reported long‐term coexistence between rats and several lizard species, with no detectable effect on the occurrence of endemic reptiles, even on small islands (Escoriza 2020). These findings suggest that prolonged sympatry may reduce or buffer direct negative impacts. Similarly, studies from the western Mediterranean indicate that lizards co‐occurring with rats may exhibit behavioral or demographic adjustments, including shifts in activity patterns, trophic niche, or changes in population structure, rather than experiencing immediate local extinction (Delaugerre et al. 2019; Nunes et al. 2022). Indeed, in predator–prey or competitive systems, niche segregation is a common mechanism facilitating coexistence (Santos et al. 2019; Garvey et al. 2022). However, evidence from other islands suggests that rats still show some predatory pressure on endemic reptiles. For example, a recent study from the Canary Islands detected remains of a critically endangered lizard in nearly 15% of *R. rattus* fecal samples and found that higher rat densities were associated with stronger declines in lizard populations (López‐Darias et al. 2024).

Studies examining rat–lizard interactions on islands at the global scale remain relatively scarce, and those that do exist are primarily focused on changes in lizard ecology, including abundance, behavior, diet, or habitat use, depending on the presence or absence of rats (Towns et al. 2007; Delaugerre et al. 2019; Donihue et al. 2021; Nunes et al. 2022; López‐Darias et al. 2024). To our knowledge, only one study has examined lizard behavior, morphology, and spatial niche use in relation to rat presence on Mediterranean islands (Delaugerre et al. 2019), while broader assessments on islands have mainly addressed patterns of species occurrence rather than direct ecological interactions (Escoriza 2020). Integrative assessments combining population density estimates with spatial and temporal niche analyses remain lacking. Consequently, it remains unclear whether rat–lizard coexistence reflects niche segregation, density‐dependent interactions, behavioral adjustments, or a combination of these processes. To address this gap, we conducted an integrative study combining population density estimation with analyses of spatial and temporal niche use on a small uninhabited Mediterranean island where *R. rattus* and the native Italian wall lizard (*Podarcis siculus*) co‐occur. To better understand this system, we aimed to assess the spatial and temporal niches of other terrestrial vertebrates that may occur on the island. Rather than assuming negative effects of rats on lizard populations, we build our hypothesis on the long‐term coexistence of those two species in the Mediterranean area.

**FIGURE 1:**
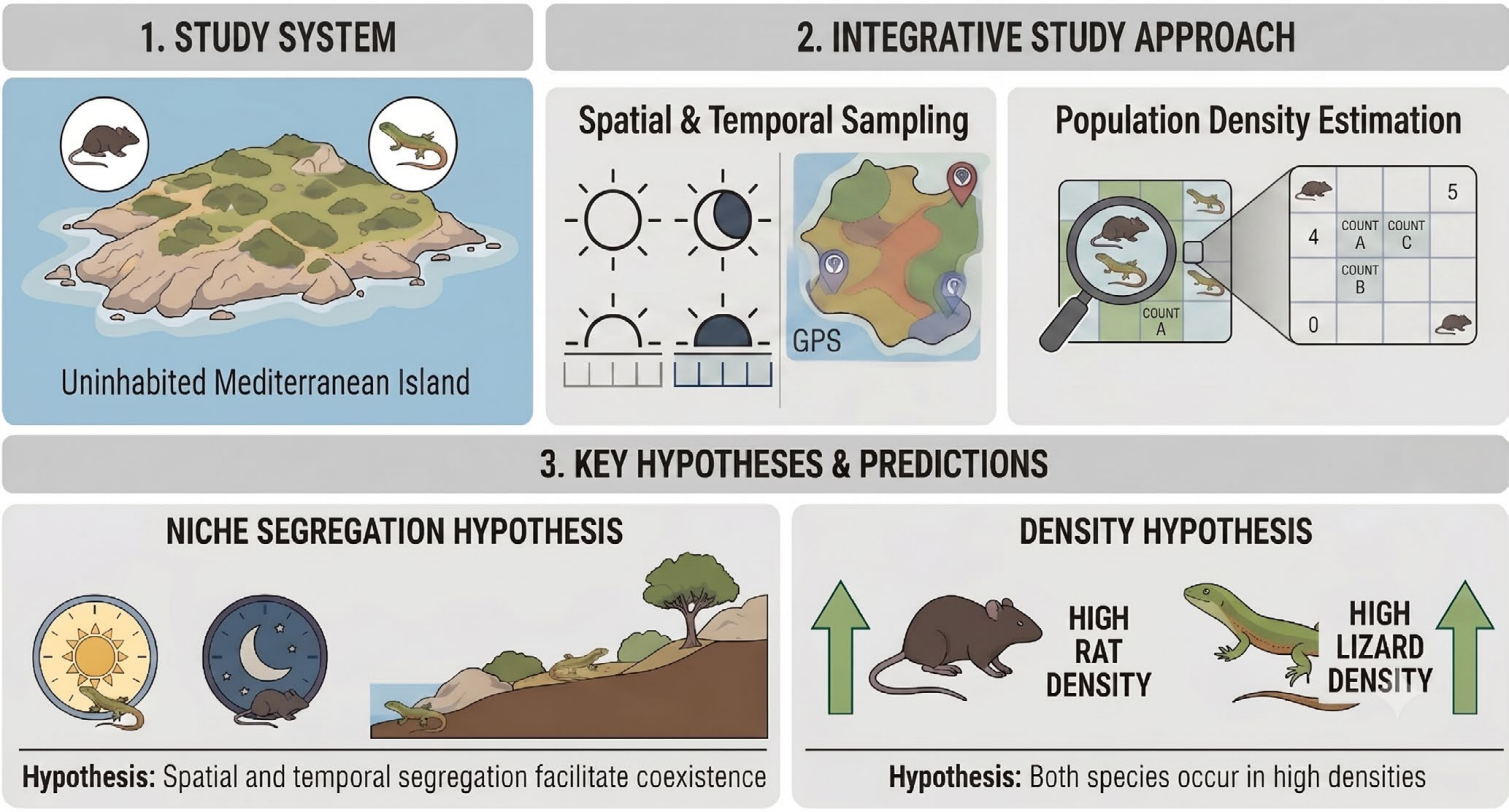
Graphical representation of design, prediction and hypothesis of our research. The figure is partially generated by AI.

We evaluated two potential consequences of species coexistence: differences in habitat use and activity patterns, and changes in overall lizard population densities. We hypothesized that if niche partitioning facilitates coexistence, *R. rattus* and *P. siculus* would exhibit clear spatial and temporal segregation. By reducing the potential for direct interspecific interactions, such segregation should allow both species to occur at relatively high densities comparable to those generally reported across other Mediterranean islands (Figure 1).

## 2 Materials and Methods

### 2.1 Study Area and System

**FIGURE 2:**
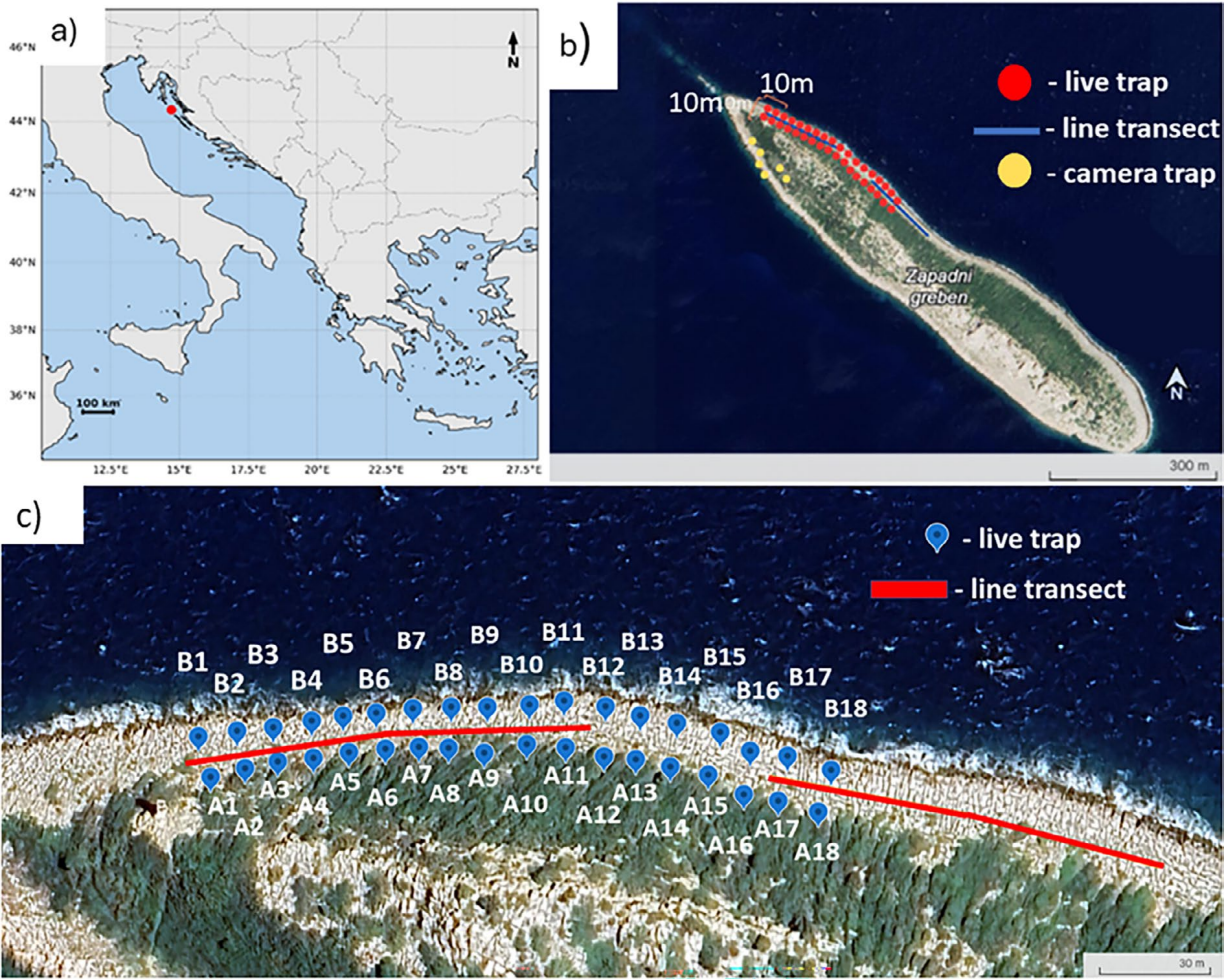
(a) Location of the Silba Reefs in the Adriatic Sea (red dot). (b) Schematic representation of the position of single‐catch live traps, line transects and camera traps on the Western Reef. Live traps are shown as red dots, transects are shown as blue lines and camera traps are shown as yellow dots. The figure is based on a Google Earth background. (c) Enlarged view of the study area on the Western Reef showing the spatial arrangement of single‐catch live traps (blue markers) and line transects (yellow lines). The figure is based on a Google Earth background.

**FIGURE A1:**
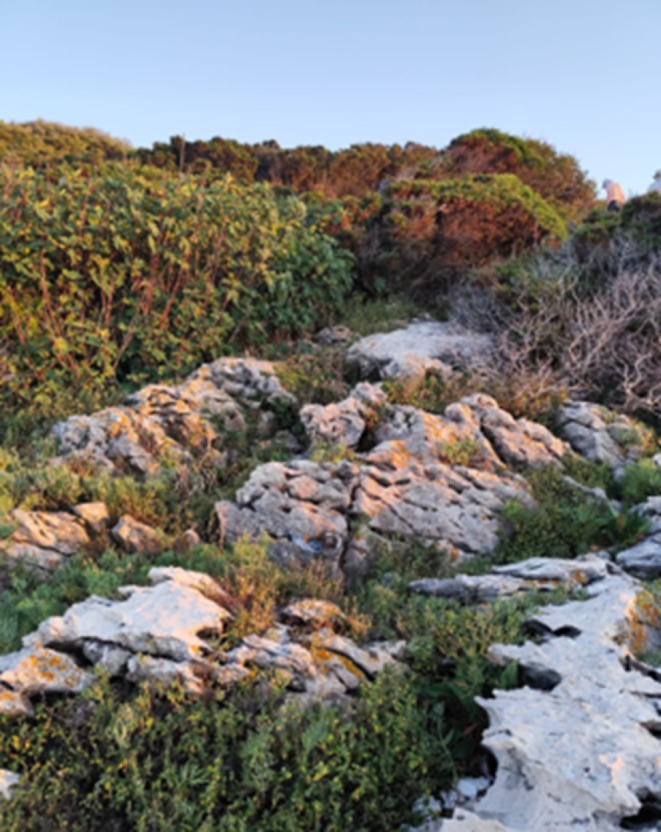
Typical maquis habitat on the western islet of the Silba Reefs.

**FIGURE A2:**
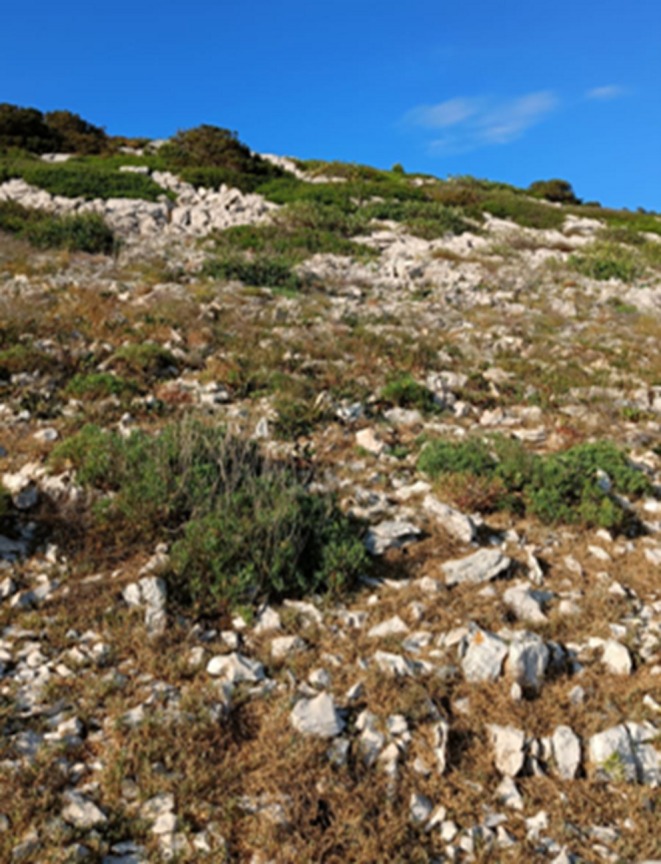
Typical dry Mediterranean grassland habitat on the western islet of the Silba Reefs.

**FIGURE A3:**
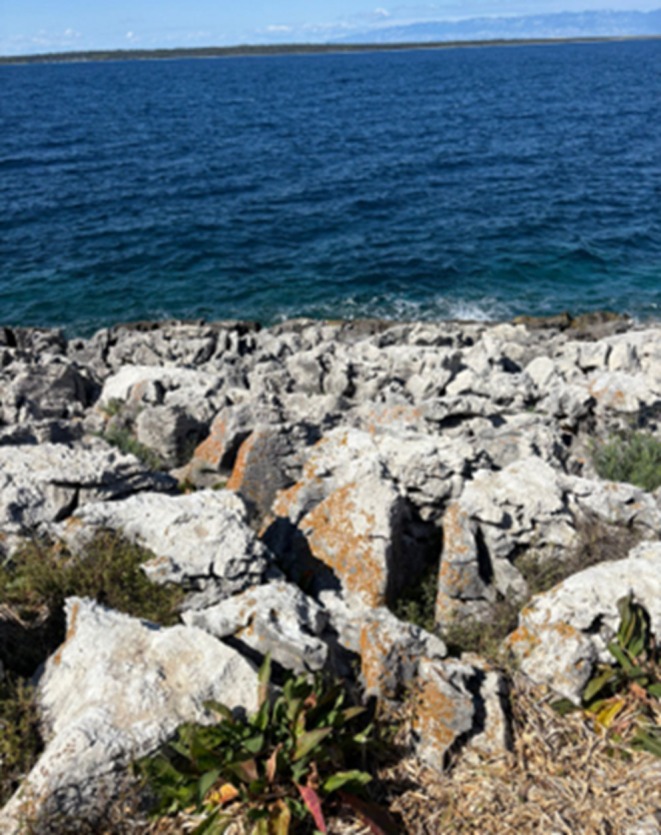
Typical rocky habitat on the western islet of the Silba Reefs.

The study took place in the Silba reef system in the northern Adriatic Sea, Croatia (Figure 2A), a Natura 2000 site (HR4000025) composed of three small uninhabited limestone islets characterized by rocky coastlines, Mediterranean shrub vegetation (maquis), and patches of dry grassland. The maquis habitat is primarily composed of *Pistacia lentiscus* and *Phillyrea latifolia* (Figure A1). Grassland habitats were characterized by dry Mediterranean grassland vegetation (Figure A2), including *Koeleria splendens*, whereas rocky coastal habitats supported sparse halophytic vegetation, including *Limonium* spp. and *Crithmum maritimum* (Figure A3). The surrounding marine area includes extensive *Posidonia oceanica* meadows, and the islets host one of the largest breeding colonies of European shags (*Gulosus aristotelis desmarestii*) in the Adriatic Sea (JU Natura Jadera 2022).

Fieldwork was carried out for five consecutive days in July 2025, following the seabird breeding season and during peak activity of terrestrial vertebrates. Sampling was carried out on the northern slope of the western islet in the reef system. Both *R. rattus* and *P. siculus* are present on all three islets, and the western islet was selected because of logistical accessibility and the feasibility of systematic sampling. To our knowledge, no previous systematic assessment of terrestrial vertebrate fauna has been conducted on these islets.

The study system represents a simplified insular terrestrial community lacking native mammalian terrestrial predators, in which *R. rattus* and *P. siculus* are supposed to be the dominant terrestrial vertebrates. Such conditions provide a suitable model system for examining coexistence dynamics and spatial–temporal niche relationships between the two species. Specifically, we quantified population density of both species and their spatial and temporal niche use by utilizing a combination of live trapping, line transects, and camera trapping.

All procedures were conducted in accordance with applicable animal welfare regulations and ethical standards for field research and were approved by the Ethical Committee of the Department of Biology, Faculty of Science, University of Zagreb (Class: 643‐02/25‐01/17; No: 251‐58‐1061725‐416). Sampling did not involve the euthanasia of animals, and all captured ship rats were released near the capture site after marking. No additional permits from the Croatia Ministry of Environment and Energy were required because the studied species and populations are not strictly protected in Croatia.

### 2.2 Genral Biology of *R. rattus* and *P. siculus*

*Rattus rattus* is a highly adaptable rodent originally native to the Indian subcontinent. It is characterized by its large, hairless ears and a tail that is notably longer than its body. Through centuries of human global travel and maritime trade, it has achieved a near‐ubiquitous worldwide distribution, establishing populations on every continent except Antarctica and becoming the most common invasive rodent on ocean islands. *Rattus rattus* is an agile, arboreal climber that thrives in a vast array of habitats, ranging from dense urban centers and agricultural landscapes to uninhabited islands and islets. While technically omnivorous, its diet is primarily frugivorous and granivorous, though it opportunistically preys on insects, bird eggs, and small vertebrates. Because of its prolific reproductive rate and generalist diet, it is considered one of the world's most damaging invasive species, heavily implicated in the decline of native island fauna, widespread agricultural damage, and the transmission of zoonotic diseases (Nowak 1999).

*Podarcis siculus* is a highly polymorphic and robust species belonging to Lacertidea. While typically characterized by a green or brownish back with darker longitudinal stripes or reticulations, the species exhibits immense local variation, most notably in small island populations where it frequently develops striking melanism (dark coloration) or unique blue patterning. *Podarcis siculus* is a diurnal, heliothermic generalist that occupies an exceptionally broad ecological niche. It flourishes equally well in natural Mediterranean macchia, agricultural edges, and heavily urbanized human settlements. While its baseline diet is strictly insectivorous, it demonstrates remarkable ecological plasticity. In addition, specific island populations have been documented undergoing rapid evolutionary shifts to incorporate significant amounts of plant matter into their diets, even developing specialized intestinal structures (cecal valves) to digest cellulose. The species is native to Italy, the major Tyrrhenian islands (Sicily, Sardinia, Corsica), and the eastern Adriatic coastline and many of its islands and islets. However, due to its generalist nature and tendency to stow away in agricultural cargo and building materials, it has become a prolific invasive species (Arnold and Ovenden 2002).

### 2.3 Estimation of Rat Density and Population Size

*Rattus rattus* were captured using 36 custom‐made metal single‐catch live traps measuring 22.5 × 7 × 8.5 cm, with mesh openings of 1.4 × 1.2 cm, arranged in two parallel lines along the northern slope of the western islet. Each line consisted of 18 traps spaced 10 m apart, with 8–10 m between the lines. The upper transect extended into the edge of maquis, while the lower transect was positioned closer to the rocky shoreline, thereby covering a gradient of habitat types (Figure 2B,C).

Traps were baited with peanut butter placed on apple slices and set in the late afternoon, at least 30 min before sunset. Traps were checked daily shortly after sunrise. Captured individuals were individually marked by shaving a small patch of fur using a beard trimmer to allow identification upon recapture. Each individual was assigned a unique ID code and released at the point of capture. Capture histories and trap coordinates were used to estimate population density with a spatially explicit capture–recapture (SECR) model.

### 2.4 Estimation of Lizard Density and Population Size

Population density of *P. siculus* was estimated using line‐transect distance sampling following protocol from Raia et al. (2010). This well‐established herpetological method (Kacoliris et al. 2009; Raia et al. 2010; Ruiz de Infante et al. 2014) relies on both the number of detections and their perpendicular distances from the transect line. These data are employed to model the decline in detection probability as distance from the observer increases, thereby allowing for robust density estimation. Two transects (100 m each) were established on the northern slope of the western islet, approximately 50 m apart and positioned at similar distances from the shoreline. Transects were placed along transitional zones between maquis and rocky habitats to encompass the main habitat types while allowing consistent observer movement (Figure 2B).

Transects were walked daily by a single observer between 08:00 and 09:00 over five consecutive days in July 2025. Surveys were conducted during a period of high lizard activity in summer while avoiding reduced detectability associated with midday heat to improve the accuracy of line‐transect density estimates. During each survey, all observed individuals were recorded, and the perpendicular distance from the transect line to each lizard was measured. Surveys were conducted at a slow and constant pace to ensure consistent detection probability. Distance data were used to estimate detection functions and calculate population density and size.

### 2.5 Camera Trapping and Niche Analysis

Six camera traps (Cuddeback H20 MP IR, Model H‐1453) were deployed on the western islet to quantify spatial and temporal activity patterns of the focal species, but also all other terrestrial vertebrates present. The cameras were distributed across three habitat types (maquis, grassland, and rocky habitat), with two cameras placed in each habitat type (Figure 2B). Cameras were mounted 20–30 cm above ground level and oriented towards animal trails or openings in vegetation to maximize the field of view and detection probability. Each camera was positioned to represent the structural characteristics of its respective habitat type while avoiding physical obstructions and was configured to record 10‐s video clips upon detection of animal movement. A 15‐s quiet period was set between recordings, so after each video the camera waited 15 s before it could be triggered again (Meek et al. 2014). Cameras were installed at the beginning of the field survey, at the start of transect sampling, and operated continuously for approximately 1 month.

All recorded terrestrial vertebrates (mammals, reptiles, and amphibians) were identified. For each detection event, species identity, date, time, and camera location were recorded. Consecutive detections of the same species within 30 s were treated as a single independent event.

### 2.6 Statistical Analysis

All statistical analyses were performed in R (R Core Team). *Rattus rattus* density was estimated using an SECR framework implemented in the package *secr* (Efford 2022). Individual capture histories and trap coordinates were used as input. A half‐normal detection function was fitted under a conditional likelihood approach (CL = TRUE), which is appropriate for single‐catch traps (Efford et al. 2009). The model estimated two key parameters: baseline detection probability (*g*_0_) and the spatial scale of movement (*σ*). A habitat mask was generated around the trap array using a buffer of 100 m, corresponding to approximately 4*σ*, which is the minimum recommended buffer width for reliable density estimation (Efford 2004). To confirm that the density estimate was insensitive to buffer choice, we conducted a buffer sensitivity analysis by fitting the model across buffer widths ranging from 25 to 200 m. Population density stabilized at buffers ≥ 100 m, confirming the adequacy of the selected buffer (Table A1). Only a single detection function (half‐normal) was fitted because the dataset (52 individuals, 83 detections across 5 occasions) was insufficient for reliable comparison among multiple detection functions.

Population density of *P. siculus* was estimated using conventional distance sampling (CDS) implemented in the R package *Distance* (Miller et al. 2019). Perpendicular distance data were pooled across five survey days and two transects. Distances were right truncated at the 95th percentile (4.17 m). Candidate detection functions included uniform, half‐normal, and hazard‐rate key functions, each with cosine and Hermite polynomial adjustment terms. We also evaluated the effect of environmental covariates (ambient temperature, wind speed, and cloud cover) on detectability using a likelihood ratio test. Temperature and wind speed data were obtained from the Croatian Meteorological and Hydrological Service (DHMZ) before each transect survey. Cloud cover was estimated visually in the field and recorded as percentage cover at the beginning of each survey. Model selection was based on Akaike's information criterion (AIC), and goodness‐of‐fit was assessed using chi‐square tests (Table A2; Appendix section “Model Selection and Covariate Testing”). Population abundance estimates for both *R. rattus* and *P. siculus* (individuals ha^−1^) were extrapolated to the total surface area of the western islet.

Diel activity patterns were modeled using kernel density estimation, and circular statistics were computed using the R packages *activity* (Rowcliffe et al. 2014), *overlap* (Ridout and Linkie 2009), and *circular* (Agostinelli and Lund 2023). Mean activity times and the mean resultant length (*r*) were calculated for each species. Rayleigh's test of uniformity was used to assess whether activity patterns deviated significantly from a uniform distribution, and Watson–Wheeler tests were employed to compare pairwise differences in activity distributions among species.

Temporal overlap between species pairs was quantified using the coefficient of overlapping (Δ_1_), which is recommended when the smaller sample in each comparison contains fewer than 50 independent detections (Ridout and Linkie 2009). Confidence intervals for Δ_1_ were obtained using 10,000 bootstrap resamples. Spatial niche use was evaluated by comparing detection frequencies among habitat types.

## 3 Results

### 3.1 Rat Density and Abundance

**FIGURE 3:**
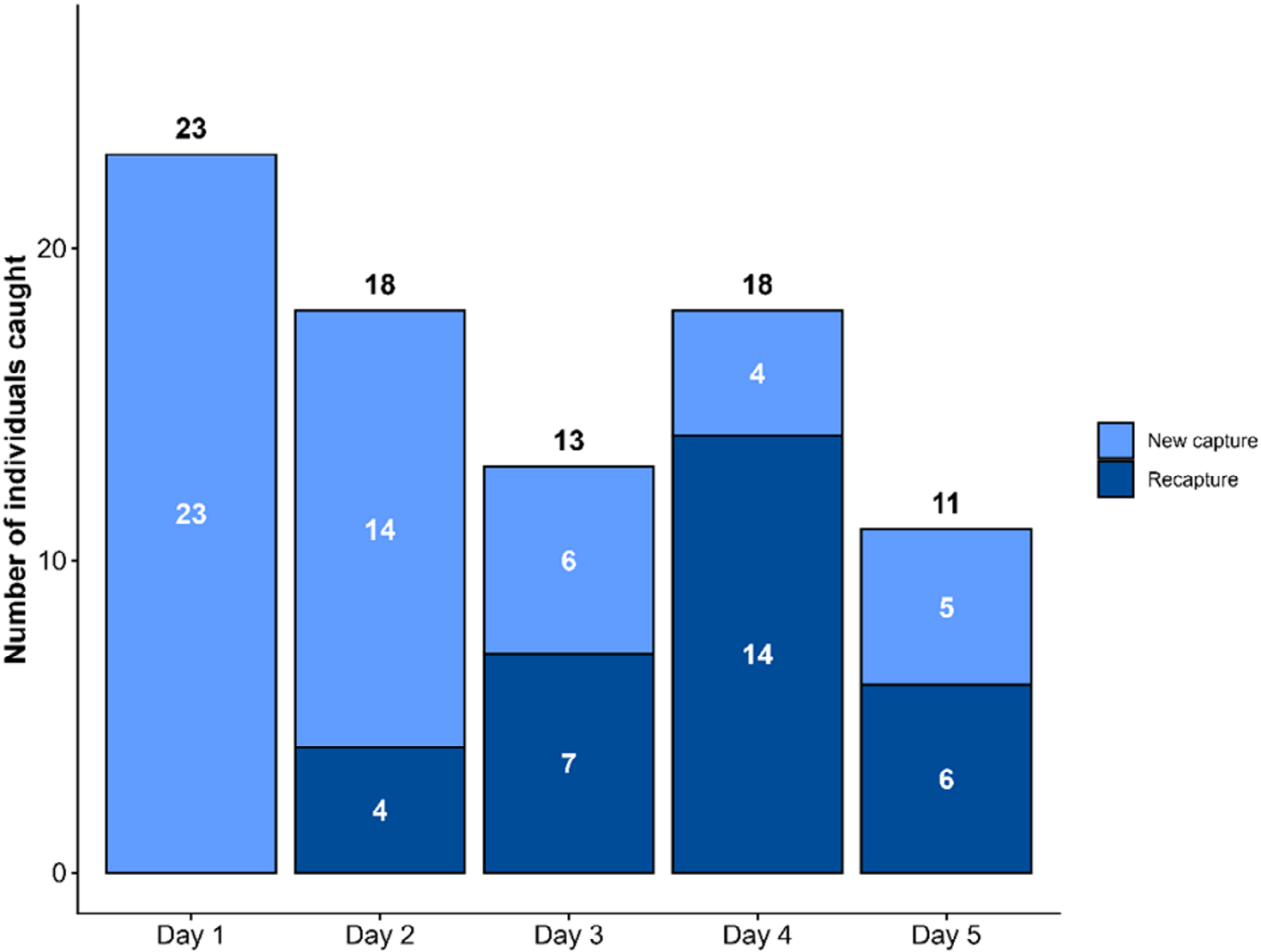
Daily number of captured ship rats ( *Rattus rattus* ) during the 5‐day trapping survey. Bars show the total number of individuals captured each day, divided into new captures (light blue) and recaptures (dark blue).

During five consecutive trapping nights (36 traps per night; 180 trap nights in total), 83 captures of *R. rattus* were recorded, including 52 unique individuals and 31 recaptures (Figure 3). Twenty individuals were captured at least twice, and the maximum number of recaptures for a single individual was four. The sample consisted of 31 males and 21 females.

The SECR model with a half‐normal detection function estimated baseline detection probability at *g*_0_ = 0.042 (SE = 0.010; 95% CI: 0.026–0.067) and the spatial scale parameter at *σ* = 24.9 m (SE = 3.75; 95% CI: 18.6–33.4). The effective sampling area (ESA) was 1.62 ha. Derived population density was estimated at 32.1 individuals ha^−1^ (SE = 7.9; 95% CI: 19.9–51.7; CVD (coefficient of variation of the density estimate) = 0.25). The density estimate was robust to buffer width selection, stabilizing at buffers ≥ 100 m (see Table A1). Assuming homogeneous density across the entire western islet (13.3 ha), total abundance was estimated at approximately 427 individuals (95% CI: 265–687).

### 3.2 Lizard Density and Abundance

**FIGURE 4:**
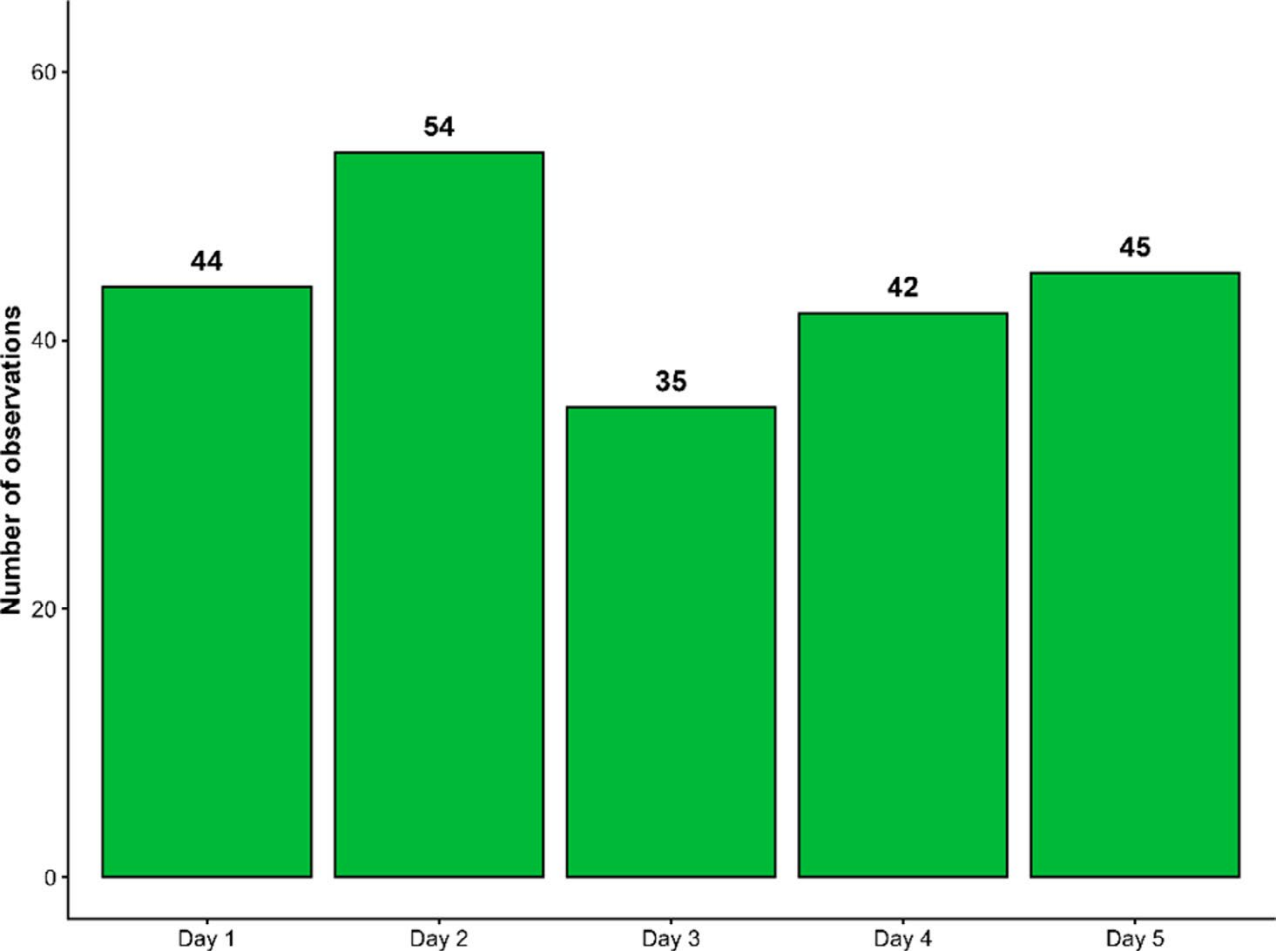
Total number of Italian wall lizards ( *P. siculus* ) individuals observed per day during five consecutive days on the same transects. Surveys were conducted under comparable weather conditions between 08:00 and 09:00 h.

The average number of *P. siculus* observed per transect per day was 44 individuals (Figure 4). The inclusion of environmental covariates (temperature, wind speed, and cloud cover) did not significantly improve model fit (likelihood ratio test: *χ* ^2^ = 1.87, df = 3, *p* = 0.599) and they were therefore excluded. Following data truncation at 4.17 m (*n* = 209 observations), the uniform key function with a cosine adjustment provided the best fit to the distance data (AIC = 512.44; Table A2). Goodness‐of‐fit tests indicated adequate model fit (chi‐square test: *p* = 0.142; Cramér–von Mises test: *p* = 0.213). The conditional detection probability was estimated at 0.534 (95% CI: 0.501–0.569), corresponding to an effective strip width of 2.23 m. *Podarcis siculus* density was estimated at 2345 individuals ha^−1^ (95% CI: 2110–2607). This density highlights a highly abundant insular population, equating to approximately one lizard for every 4.3 m^2^ of the islet surface area. Extrapolated to the entire islet (13.3 ha), total abundance was estimated at 31,189 individuals (95% CI: 28,059–34,668).

### 3.3 Camera Trap Records, Spatial and Temporal Distribution

Camera traps recorded three terrestrial vertebrate species: *R. rattus* , *P. siculus* , and the European rabbit ( *Oryctolagus cuniculus* ). A total of 607 independent detection events were obtained, including 535 rat records, 31 lizard records, and 41 rabbit records.

**FIGURE 5:**
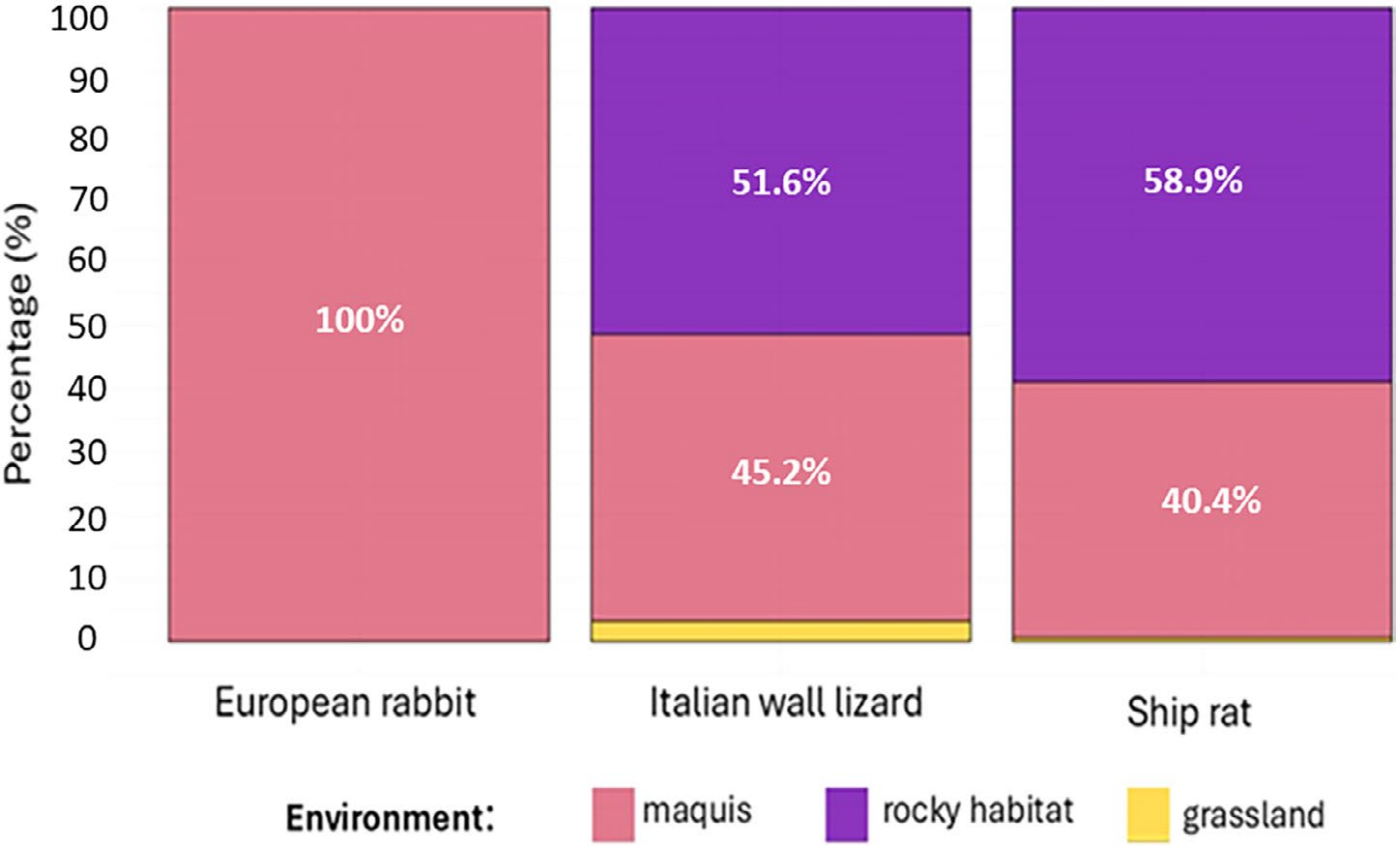
Proportional distribution of camera‐trap detections across habitat types (maquis, rocky habitat, and grassland) for ship rats ( *R. rattus* ), Italian wall lizard ( *P. siculus* ), and European rabbit ( *O. cuniculus* ).

Both *R. rattus* and *P. siculus* were detected in all three habitat types (maquis, rocky habitat, and grassland), whereas rabbits were recorded exclusively in maquis. Detection frequencies for both *R. rattus* and *P. siculus* were lowest in the grassland and relatively evenly distributed between maquis and rocky habitat (Figure 5).

**FIGURE 6:**
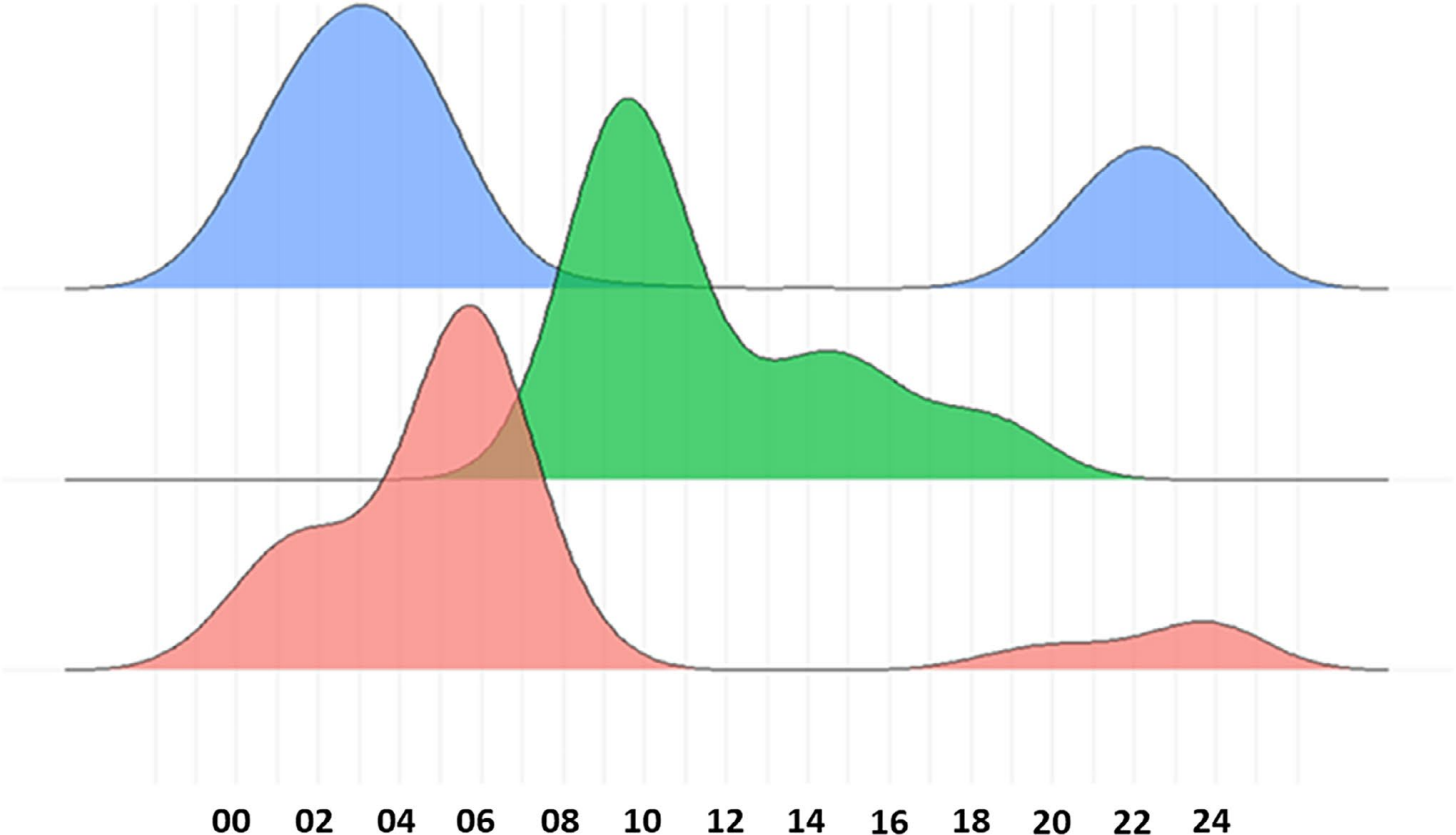
Diel activity patterns of ship rats ( *R. rattus* ) (blue), Italian wall lizard ( *P. siculus* ) (green), and European rabbit ( *O. cuniculus* ) (red) based on kernel density estimation of camera‐trap detections.

All three species exhibited strongly non‐uniform diel activity distributions (Rayleigh's test: *p* < 0.001 for all species). *R. rattus* exhibited predominantly nocturnal activity, with mean activity centered at 01:42 and a high concentration of activity (mean resultant length *r* = 0.774). Activity showed a bimodal pattern, with a primary peak around 03:10 and a secondary peak near 22:00 (Figure 6). Occasional low‐intensity daytime activity was also detected. On the contrary, *P. siculus* showed a strongly diurnal pattern, with mean activity at 11:13 (*r* = 0.718) and a primary peak around 09:50 and a secondary increase in early afternoon (14:00–15:00). On the other hand, *O. cuniculus* displayed crepuscular activity, with a dominant morning, mean activity at 04:12 (*r* = 0.753) and a peak around 05:45 and a weaker afternoon peak (Figure 6).

Watson–Wheeler tests confirmed that activity distributions differed significantly between all species pairs. The greatest difference was observed between *R. rattus* and *P. siculus* (*U* ^2^ = 2.397, *p* < 0.001), consistent with the near‐complete separation of nocturnal and diurnal activity windows. Differences between *P. siculus* and *O. cuniculus* were also pronounced (*U* ^2^ = 1.474, *p* < 0.001), whereas the comparison between *R. rattus* *and O. cuniculus* yielded the lowest test statistic (*U* ^2^ = 0.812, *p* < 0.001), reflecting partial temporal overlap during crepuscular hours.

Temporal overlap coefficients (Δ_1_) corroborated these patterns quantitatively. Overlap between *R. rattus* and *P. siculus* was extremely low (Δ_1_ = 0.029; 95% CI: 0.015–0.047), indicating near‐complete temporal segregation. Overlap between *P. siculus* and *O. cuniculus* was also low (Δ_1_ = 0.079; 95% CI: 0.031–0.140), as lizard activity ceased before the main crepuscular peak of rabbits. In contrast, overlap between *R. rattus* and *O. cuniculus* was moderate (Δ_1_ = 0.567; 95% CI: 0.431–0.730), consistent with shared activity during pre‐dawn hours.

## 4 Discussion

This study provides the first integrative assessment of population density and spatial and temporal niche use of *R. rattus* and *P. siculus* on a small Mediterranean islet. As expected, activity patterns revealed clear temporal segregation between the two species, but no spatial segregation was detected. Moreover, we recorded relatively high densities of *P. siculus* compared to similar systems, despite concurrently high *R. rattus* densities, demonstrating that these species can successfully coexist. Finally, only three terrestrial vertebrate species were detected on the islet: *P. siculus* , *R. rattus* , and *O. cuniculus*. The low number of terrestrial vertebrate species likely simplifies interspecific interactions, making this island a suitable system for examining rat–lizard coexistence.

Introduced rats are widely associated with reptile population declines on islands, particularly where rats and reptiles exhibit substantial spatial or temporal niche overlap (Towns 1991; Hoare et al. 2007; Towns et al. 2007). A recent study from the Canary Islands detected remains of the critically endangered lizard *Gallotia intermedia* in nearly 15% of ship rat fecal samples and found that higher rat densities were associated with stronger declines in lizard populations (López‐Darias et al. 2024). Several studies report increases in lizard abundance following rat removal. For example, Donihue et al. (2021) documented population increases and behavioral shifts in a Caribbean lizard after the eradication of both rats and goats. However, because rats and goats were removed simultaneously and vegetation structure subsequently changed, thepecific contribution of rat removal to lizard recovery remains difficult to disentangle from broader habitat alterations. Similarly, responses of lizards to invasive mammal removal have been documented elsewhere. On Berlenga Island (Portugal), the eradication of *R. rattus* and *O. cuniculus* was followed by an expansion of the trophic niche of the endemic lizard *Podarcis carbonelli berlengensis*, suggesting that invasive mammals can alter trophic interactions and resource availability for insular reptiles (Nunes et al. 2022). In contrast, Escoriza (2020) reported long‐term coexistence between rats and Mediterranean lizard species across numerous islands, suggesting that prolonged sympatry may buffer or reduce direct negative impacts. Our results align more closely with this latter pattern. We calculated *R. rattus* density to be approximately 32.1 individuals ha^−1^ (estimated during the presumed seasonal peak in summer). This value exceeds densities typically reported from mainland populations, which generally range from less than 1 to approximately 16.5 individuals ha^−1^ (Nordmeier 2020; Carpenter et al. 2022). However, our calculated value falls within the range reported for insular populations of *R. rattus* . For example, densities of approximately 6 to 10 individuals ha^−1^ have been reported from Ponui Island, New Zealand (Shapiro 2005), while population densities on Big South Cape Island/Taukihepa can reach 36.4 individuals ha^−1^ in late summer (Harper and Rutherford 2016). Even higher densities have been recorded on tropical islands, including 88–187 individuals ha^−1^ on Diego Garcia in the Indian Ocean (Vogt et al. 2014). Alongside this, lizard density in our study reached 2345 individuals ha^−1^, corresponding to high abundance on a small island. We haven't found any reported estimates of population density for *P. siculus* , however, this value falls within the range reported for insular lacertid populations. For instance, densities of *Podarcis lilfordi* on small Balearic islets range from fewer than 35 to nearly 8000 individuals ha^−1^ (Pérez‐Mellado et al. 2008). Similarly, densities between approximately 1300 and 3700 individuals ha^−1^ have been reported for island populations of *P. lilfordi* using line‐transect distance sampling (Ruiz de Infante et al. 2014). Extreme densities exceeding 50,000 individuals ha^−1^ have been documented in small geckos such as *Sphaerodactylus macrolepis* (Rodda et al. 2001). In contrast, mainland lacertid populations typically occur at much lower densities, often not exceeding a few dozen individuals per hectare (Maura et al. 2011).

Temporal niche segregation likely represents a key mechanism enabling coexistence in this system. Rats were predominantly nocturnal, whereas lizards were strictly diurnal with reduced activity during the hottest hours, consistent with the typical activity patterns of both species (Bauwens et al. 1996; Shiels et al. 2014; Harper and Bunbury 2015). Temporal partitioning can reduce encounter rates and limit direct interactions between species even when spatial overlap is high (Santos et al. 2019; Garvey et al. 2022). Yet this observed temporal segregation does not eliminate the possibility of occasional encounters between rats and lizards. Rats may still occasionally take lizard juveniles, eggs, or poorly sheltered individuals at night. However, the combination of strong diel separation and very high lizard density suggests that any predation is currently not limiting the lizard population on the island. Similar patterns have been observed in other island systems, where behavioral plasticity and activity shifts facilitate persistence despite invasive predator presence (Hoare et al. 2007). However, activity patterns in both rodents and lizards are known to vary in response to environmental conditions such as temperature, solar radiation, and precipitation (Zamora‐Camacho et al. 2013; Williams et al. 2014), suggesting that diel segregation observed during summer may not necessarily persist across other seasons. Therefore, replication of this research in different seasons is needed to test whether this segregation persists outside summer conditions. Spatial analyses indicated that both *R. rattus* and *P. siculus* were detected across all habitat types but were recorded most frequently in maquis and rocky habitats, indicating substantial habitat overlap. On small islands, such habitat use is not unexpected, because limited area and habitat heterogeneity often result in broad spatial overlap among terrestrial vertebrates (Pérez‐Mellado et al. 2008). Rats are known for flexible habitat use and the capacity to occupy diverse microhabitats (Harper and Bunbury 2015). On the other hand, *P. siculus* is also known for its opportunistic strategies, a characteristic that enables this species to adapt to human altered habitats and establish invasive populations (Kapsalas et al. 2016). The observed spatial overlap suggests that coexistence on the western islet of the Silba Reefs is not achieved through strong habitat partitioning. Instead, both species appear to share space extensively, reinforcing the importance of temporal rather than spatial segregation in reducing direct interactions. In small insular systems, where spatial escape opportunities are limited, temporal differentiation may represent a more efficient strategy for reducing direct interactions between species. However, the coexistence of these species on the islet may also be facilitated by differences in their trophic ecology. Although both *R. rattus* and *P. siculus* are opportunistic generalists, *R. rattus* primarily feeds on plant material, seeds, fruits, and arthropods, whereas *P. siculus* relies mainly on arthropod prey, although insular populations may also incorporate substantial amounts of plant material into their diet (Shiels et al. 2014; Taverne et al. 2019). Furthermore, the difference in body size between the two species likely results in the use of different resource and prey size classes, reducing the potential for strong trophic overlap. Together with temporal niche segregation, such dietary differences may contribute to the coexistence of both species on the islet, which have to be tested in the future. The only other terrestrial vertebrate species observed with camera trapping, *O. cuniculus* , displayed crepuscular activity and was detected primarily in maquis habitat, indicating both temporal and partial spatial differentiation from the other two species. Rabbits are well known to influence island ecosystems through grazing, often altering vegetation structure and indirectly affecting other taxa (Kossoff et al. 2024). In some island systems, rabbit population increases have had cascading effects mediated through invasive rats. For example, on Whale Island (New Zealand), a rapid increase in rabbit density was associated with increased rat abundance, likely because rabbit juveniles and carcasses provided an additional food source that sustained rat populations outside the seabird breeding season (Imber et al. 2000). In contrast, we have no evidence of rabbits currently influencing rat abundance or substantially altering vegetation structure on our study islet. The island is predominantly covered with dense maquis growing on extremely rocky terrain, which may buffer against strong grazing impacts. Given the comparatively low detection frequency of rabbits and the absence of clear vegetation degradation, their ecological role in this system appears secondary at present. Nevertheless, potential indirect interactions, particularly under different management scenarios such as rat eradication, should be considered.

Spatial analyses indicated that both *R. rattus* and *P. siculus* were detected across all habitat types but were recorded most frequently in maquis and rocky habitats, indicating substantial habitat overlap. On small islands, such habitat use is not unexpected, because limited area and habitat heterogeneity often result in broad spatial overlap among terrestrial vertebrates (Pérez‐Mellado et al. 2008). Rats are known for flexible habitat use and the capacity to occupy diverse microhabitats (Harper and Bunbury 2015). On the other hand, *P. siculus* is also known for its opportunistic strategies, a characteristic that enables this species to adapt to human‐altered habitats and establish invasive populations (Kapsalas et al. 2016). The observed spatial overlap suggests that coexistence on the western islet of the Silba Reefs is not achieved through strong habitat partitioning. Instead, both species appear to share space extensively, reinforcing the importance of temporal rather than spatial segregation in reducing direct interactions. In small insular systems, where spatial escape opportunities are limited, temporal differentiation may represent a more efficient strategy for reducing direct interactions between species. However, the coexistence of these species on the islet may also be facilitated by differences in their trophic ecology. Although both *R. rattus* and *P. siculus* are opportunistic generalists, *R. rattus* primarily feeds on plant material, seeds, fruits, and arthropods, whereas *P. siculus* relies mainly on arthropod prey, although insular populations may also incorporate substantial amounts of plant material into their diet (Shiels et al. 2014; Taverne et al. 2019). Furthermore, the difference in body size between the two species likely results in the use of different resource and prey size classes, reducing the potential for strong trophic overlap. Together with temporal niche segregation, such dietary differences may contribute to the coexistence of both species on the islet, which have to be tested in the future. The only other terrestrial vertebrate species observed with camera trapping, *O. cuniculus* , displayed crepuscular activity and was detected primarily in maquis habitat, indicating both temporal and partial spatial differentiation from the other two species. Rabbits are well known to influence island ecosystems through grazing, often altering vegetation structure and indirectly affecting other taxa (Kossoff et al. 2024). In some island systems, rabbit population increases have had cascading effects mediated through invasive rats. For example, on Whale Island (New Zealand), a rapid increase in rabbit density was associated with increased rat abundance, likely because rabbit juveniles and carcasses provided an additional food source that sustained rat populations outside the seabird breeding season (Imber et al. 2000). In contrast, we have no evidence of rabbits currently influencing rat abundance or substantially altering vegetation structure on our study islet. The island is predominantly covered with dense maquis growing on extremely rocky terrain, which may buffer against strong grazing impacts. Given the comparatively low detection frequency of rabbits and the absence of clear vegetation degradation, their ecological role in this system appears secondary at present. Nevertheless, potential indirect interactions, particularly under different management scenarios such as rat eradication, should be considered.

### 4.1 Strengths, Limitations, and Future Directions

A major strength of this study is its integrative design, which combines density estimation methods (SECR and distance sampling) with quantitative temporal and spatial niche analyses, an approach that, to our knowledge, has not been applied before in island systems in general and remains unexplored in the Mediterranean region as well. More precisely, only one study on Mediterranean islands has explicitly examined rat‐lizard interactions (Delaugerre et al. 2019), whereas other work has focused primarily on large‐scale occurrence patterns rather than direct ecological interactions (Escoriza 2020). Integrative studies combining population density estimates with spatial and temporal niche analyses are currently lacking. By focusing on a small and simplified island system, our study reduces confounding trophic interactions and provides a clearer picture of coexistence dynamics. Additionally, it offers novel insights into rat–lizard interactions in the Adriatic region, where density‐based assessments of such systems are not present.

However, several limitations must be acknowledged. First, fieldwork was conducted during a single summer season and lasted only five consecutive days. Seasonal and yearly variation in activity, reproduction, and resource availability may alter both density estimates and niche relationships. Secondly, the study was restricted to one islet. Specific characteristics of an island may strongly influence outcomes. Therefore, results should be interpreted and generalized with caution. Future research should expand to multiple islands differing in rat presence, island size, and vegetation structure. Also, comparative studies on islands before and after eradication would allow stronger causal inference. Previous eradication efforts demonstrate that removing invasive species can trigger rapid ecological and morphological shifts, such as niche expansion and increased body size in insular lizards (Jones et al. 2008; Shiels et al. 2014; Donihue et al. 2021; Nunes et al. 2022). In addition, eradication can cascade across the wider ecosystem to alter vegetation structure and improve seabird reproductive success. Should similar eradication measures be applied to our study islet, parallel ecological trajectories would be expected. Long‐term, multi‐season sampling is needed to determine whether temporal segregation remains stable year‐round. Beyond density and activity metrics, future research on Silba Reefs should integrate behavioral, morphological, and trophic data, alongside estimates of predation pressure and the population structure of all terrestrial vertebrates (including *O. cuniculus* ). Synthesizing these diverse parameters would facilitate a more comprehensive understanding of the compensatory mechanisms that permit the coexistence of *R. rattus* and *P. siculus* (Donihue et al. 2021; Nunes et al. 2022).

## 5 Conclusions

By examining an ecologically simplified Mediterranean islet, this study demonstrates how temporal niche partitioning facilitates coexistence between *P. siculus* and *R. rattus* . The relatively high densities of both species within a limited area suggest that rat presence is not currently constraining lizard population size. Pronounced temporal niche segregation between diurnal lizards and predominantly nocturnal rats likely reduces encounter rates despite extensive spatial overlap, although additional mechanisms may also contribute. These findings improve our understanding of rat–lizard interactions under long‐term sympatry in Mediterranean island systems. Rather than resulting in local extinction, prolonged coexistence may promote ecological adjustments that enable native lizard populations to persist even in the presence of invasive rats.

## Author Contributions

**Maja Perinović:** conceptualization (equal), data curation (equal), formal analysis (equal), investigation (equal), methodology (equal), validation (equal), visualization (equal), writing – original draft (equal), writing – review and editing (equal). **Tomislav Gojak:** conceptualization (equal), data curation (equal), investigation (equal), methodology (equal), software (lead), validation (equal), visualization (equal), writing – review and editing (equal). **Marko Glogoški:** conceptualization (equal), investigation (equal), methodology (equal), validation (equal), visualization (equal), writing – review and editing (equal). **Luce Pavin:** investigation (equal), writing – review and editing (equal). **Hrvoje Čižmek:** funding acquisition (equal), methodology (equal), resources (equal), writing – review and editing (equal). **Duje Lisičić:** conceptualization (lead), funding acquisition (equal), investigation (equal), methodology (equal), project administration (lead), resources (equal), supervision (lead), writing – original draft (equal), writing – review and editing (equal).

## Funding

Work on this paper was supported by project 106‐F25‐00051 “Duje Lisičić. Potpore istraživanjima 2025” (No. 20286792) and by MAPA project (https://www.italy‐croatia.eu/web/mapa) under Italy Croatia Cross Borders Cooperation Programme co‐financed by European Regional Development Fund (ERDF) and Office for Cooperation with NGOs of Government of Croatia. Authors are grateful to Marine Explorers Society—20000 Leagues for their invaluable help in field activities.

## Ethics Statement

All procedures were conducted in accordance with applicable animal welfare regulations and ethical standards for field research and were approved by the Ethical Committee of the Department of Biology, Faculty of Science, University of Zagreb (Class: 643‐02/25‐01/17; No: 251‐58‐1061725‐416). Sampling did not involve the euthanasia of animals, and all captured ship rats were released near the capture site after marking. No additional permits were required because the studied species and populations are not strictly protected in Croatia.

## Conflicts of Interest

The authors declare no conflicts of interest.

## Data Availability

The datasets generated and analyzed during the current study are available for peer review via a private, temporary Figshare link: https://figshare.com/s/03f50e7d836b3c5a5e98. Upon acceptance of this manuscript, the dataset will be published and made permanently accessible to the public on the Figshare repository under a unique https://doi.org/10.6084/m9.figshare.32969186.

## Appendix A

**TABLE A1:** Buffer sensitivity analysis for SECR.

Buffer (m)	Mask area (ha)	*D* (ind ha^−1^)	SE	95% CI
25	0.01	59.92	9.77	43.62–82.30
50	0.03	35.46	7.09	24.06–52.27
75	0.05	32.26	7.77	20.26–51.39
**100**	**0.08**	**32.07**	**7.92**	**19.90–51.66**
125	0.11	32.06	7.93	19.89–51.68
150	0.14	32.06	7.93	19.88–51.68
175	0.19	32.06	7.93	19.88–51.68
200	0.23	32.06	7.93	19.88–51.68

*Note:* Density stabilizes at buffers ≥ 100 m (≈4*σ*). Bold row indicates the selected buffer.

**TABLE A2:** Candidate models fitted to perpendicular distance data for *Podarcis siculus*.

Model	Key function	Adjustment	Covariates	AIC	∆AIC	*w*_*i*_
Unif + cos	Uniform	Cosine	None	**512.44**	**0.00**	**0.285**
HN	Half‐normal	None	None	513.25	0.81	0.190
HN + cos	Half‐normal	Cosine^a^	None	513.25	0.81	0.190
HN + herm	Half‐normal	Hermite^a^	None	513.25	0.81	0.190
HR	Hazard‐rate	None	None	515.54	3.10	0.061
HR + cos	Hazard‐rate	Cosine^a^	None	515.54	3.10	0.061
HN + cov	Half‐normal	None	Temp, wind, cloud	517.38	4.94	0.024

*Note:* Models are ranked by Akaike's information criterion (AIC). ∆AIC represents the difference in AIC relative to the top‐ranked model, and *w*_*i*_ represents the AIC model weight. The bolded entry denotes the top‐ranked model.

^a^ For these models, the adjustment terms did not improve the model fit during the stepwise selection process, causing the algorithm to default back to the base key function. Hence, their AIC values are identical to the unadjusted base models.

### Model Selection and Covariate Testing

To determine the most appropriate detection function for *P. siculus* , we fitted seven candidate models to the truncated perpendicular distance data. We evaluated uniform, half‐normal, and hazard‐rate key functions, both with and without series adjustments (cosine and Hermite polynomials). Model selection was based on Akaike's information criterion (AIC). The uniform key function with a cosine adjustment provided the most parsimonious fit to the data (AIC = 512.44) and carried the highest model weight (*w*_*i*_ = 0.285).
